# Automated image analysis method for oil-release test of lipid-based materials

**DOI:** 10.1016/j.mex.2021.101447

**Published:** 2021-07-10

**Authors:** Fabio Valoppi, Petri Lassila, Ari Salmi, Edward Haeggström

**Affiliations:** a Electronics Research Laboratory, Department of Physics, University of Helsinki, Gustaf Hällströmin katu 2, PO Box 64, Helsinki 00014, Finland; b Department of Food and Nutrition, University of Helsinki, Agnes Sjöbergin katu 2, PO Box 66, Helsinki 00014, Finland; c Faculty of Agriculture and Forestry, Helsinki Institute of Sustainability Science, University of Helsinki, Helsinki 00014, Finland

**Keywords:** Oil binding capacity, Filter paper, ImageJ, Oleogels, Fats

## Abstract

We present an automated method to determine oil release from lipid-based materials. Oil-release tests can provide information regarding the ability to retain oil within the structuring network of lipid-based materials. This test provides a first insight into the stability of these materials and their possible applications in food, cosmetic, and pharmaceutical products. The method presented features a simple setup comprising a camera that automatically captures images of the evolution of the oil stain released from lipid-based materials placed on a filter paper. Image postprocessing is performed with two custom-made scripts developed for the freeware application ImageJ. The scripts allow direct calculation of the oil-stain area from all images stored in a folder returning as output numerical values in a table. This method was shown to be:

• inexpensive, as the employed tools and equipment are available in most laboratories both in academia and industry,
• self-running, as the method automatically captures images at predefined time intervals for a certain time span,
• practical, as manual-image analysis is unnecessary (200 images can be automatically analyzed in 3 min).

## Graphical abstract

**Figure:**
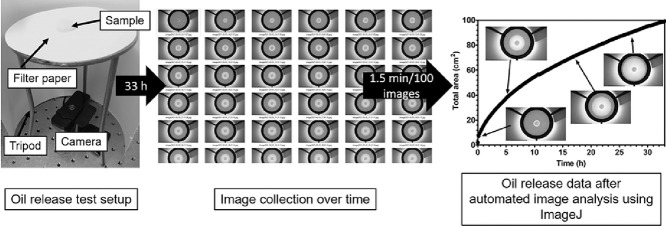

Specifications table

**Table:** 

Subject Area:	Materials Science
More specific subject area:	*Food Engineering*
Method name:	Image analysis for oil-release test
Name and reference of original method:	Rogers, M. A., Wright, A. J., Marangoni, A. G., Engineering the oil binding capacity and crystallinity of self-assembled fibrillar networks of 12-hydroxystearic acid in edible oils. *Soft Matter.* 2008, *4*, 1483-1490. This paper describes an oil-release test on filter paper where the filter paper is weighed at after 1 h of analysis. No time-dependent data were collected with this method.
Resource availability:	YawCam - https://www.yawcam.com/ ImageJ - https://imagej.nih.gov/ij/

## Background

Lipid-based materials (LBM), such as mixtures of structuring molecules (including solid fats, emulsifiers, carbohydrates) and oils are widely used in food, cosmetic, and pharmaceutical products. Such products include spreads, creams, lotions, and drug and bioactive molecules delivery systems [1], [2], [3]. As LBMs are mainly gelled systems due to the 3D network of structuring molecules that retains oil, they confer texture and functionality when used as ingredients. Unfortunately, oils in LBMs are mobile and can migrate out from the network [4]. This phenomenon leads to oil leaking and jeopardizes the functionality and acceptance of products containing LBMs. Consequently, oil-retention ability is an important parameter that should be evaluated in LBMs.

Oil-retention ability (also oil-binding capacity [OBC]) is mainly determined using centrifugation or filter-paper methods [5]. The first method is based on centrifugal forces that expel oil out from the sample. This method is referred to as accelerated oil-release analysis, as it can take a maximum of 30 min for each sample and several samples can be analyzed in a single run. However, the expulsion of oil from LBM during centrifugation is based on differences in densities between the oil and on the network of structuring molecules. In addition, centrifugation can deform and damage the network, making comparisons among different LBM challenging [5]. Filter-paper methods measure the weight increment of the filter paper where the LBM is placed. Oil is pulled out from the network by capillary forces, the LBM is removed from the filter paper, any sample residuals sticking to the paper are removed, and finally the paper is weighed [4,6]. Unfortunately, this method is labor intensive and requires several parallel samples.

We developed an automated analysis based on image analysis for determining oil release on filter paper. We used a simple setup featuring a tripod with an opening on top and a camera that captures images at regular intervals of the growing oil stain that migrates from the LBM to the filter paper. We then developed two scripts for the freeware, open-source image analysis application software ImageJ. The scripts perform an automated image analysis returning the output area (cm^2^) of the oil stain in each captured image. The time-lapse values are plotted as a function of time to obtain the oil release of LBM. This paper introduces a new automated method for determining the oil release from LBM, which can be potentially extended to other materials formed by a mobile non-volatile solvent trapped in a network.

## Setup

**Fig. 1:**
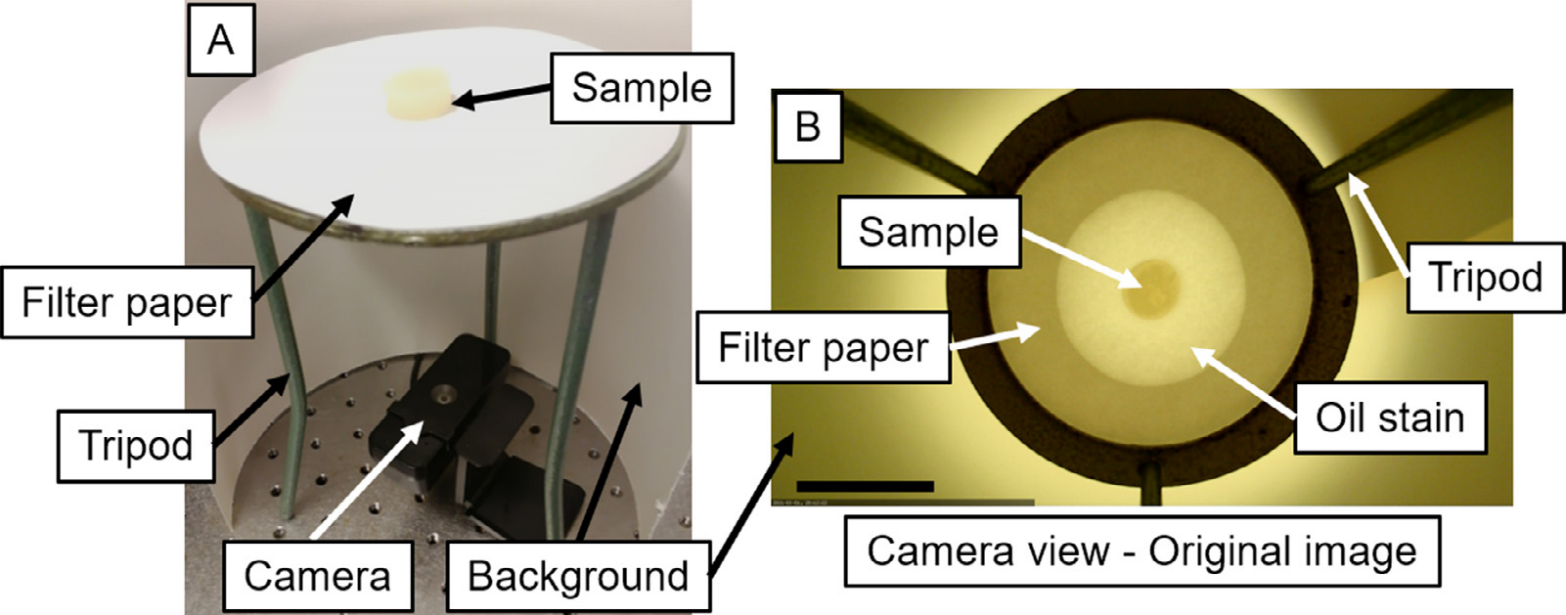
(A) Oil-release setup featuring a digital HD camera fixed to an optical table, a tripod with a circular opening on top, a filter paper disk, and the sample placed on the filter paper. (B) Camera view showing the circular opening on the tripod and an oil stain forming from the sample placed at the center of the filter paper. The background in (B) was obtained using a cylinder of thick paper. Scale bar in (B) is 5 cm.

We built a simple setup to automatically image over time the evolution of an oil stain obtained by laying LBM on filter paper (Fig. 1A). The setup features a tripod with a circular opening on top (tripod height: 21.5 cm, opening diameter: 12.1 cm), a full HD digital camera (Lenovo essential FHD webcam) fixed on an optical table pointing upwards (the camera aperture must be perpendicular to the optical table), and a laptop running the freeware application YawCam (v.0.7.0, https://www.yawcam.com/). The software captures and automatically saves the pictures at pre-selected time intervals. In this example, images were taken every 10 min. Images were saved in a temporary folder of Windows and should be transferred to the destination folder after completion of analysis. A 10% monoglyceride-based oleogel was selected here as an example of LBM (more information about the oleogel preparation procedure can be found in [7]). After preparation, the oleogel was cut into cylinders (diameter: 20 mm, height: 7-10 mm, mass: 3.25 ± 0.5 g) using a metallic cylindrical shaper with thin walls and was placed on a filter-paper disk (Whatman filter paper #2 disk, material: cellulose, pore size: 8 µm, thickness: 190 µm, diameter: 15 cm), which was then placed on the tripod. A paper cylinder (height: 75 cm, diameter: 20 cm, paper thickness: 0.4 cm, total thickness of paper after multiple folds: 1.2 cm) was used to isolate the setup from the external environment and provided a light background. Illumination was achieved by a commercial neon tube light positioned 1 m above the setup, whose light diffused through the paper cylinder used as background. Light intensity has to be kept constant thought all the analyses to ensure consistency and comparability between the results. This can be achieved by maintaining the setup in the same position with respect to the light source. Moreover, script #2 (see below) uses a function that reduces the effect of possible light fluctuation. Using graph paper, we determined that the portion of the picture where the filter paper was located was optically flat and therefore did not introduce optical distortions during image analysis. Moreover, the mass of the sample placed on the filter paper was sufficiently small (below 4 g) to avoid bending the filter paper and creating distortions in the acquired images. Preliminary experiments were necessary to select the proper filter paper (*i.e.,* pore diameter, disk diameter, disk thickness, and material) and sample weight, and dimensions, such that the filter paper efficiently absorbed oil from the LBM *via* capillary forces, it allowed the formation and growth of the oil stain within the selected time frame of the analysis, there was no solid material transfer between the LBM and the filter paper (absorption of LBM network in the filter paper), and no bending of the filter paper due to the weight of the sample can be observed during the analysis. The distance between the camera and the bottom of the filter paper was 15 cm. The automatic image capturing application software was then started. An example of a captured image is shown in Fig. 1B. Image collection was manually stopped after the pre-selected amount of time elapsed (33 h in this specific example). Experiments were performed at room temperature (22 ± 1°C). However, to obtain reliable and comparable measurements, the temperature needs to be controlled and kept constant among experiments because the rate of oil release in LBM is affected by temperature.

## ImageJ scripts

We developed two scripts for ImageJ application software (macro files in supplementary material). The latter is a freeware, open-source software for image analysis (https://imagej.nih.gov/ij/) [8]. These scripts automatically analyzed all images collected in a single folder, acquired using the setup described above, to obtain a table with numerical values that can be used to plot the oil release of LBM as a function of time. After downloading the macro files (supplementary material), they should be modified to add the correct folder full paths of images to be analyzed (*i.e.,* C:/Users/…/). This can be done in ImageJ by selecting Plugins → Macros → Edit… and opening the downloaded files. In addition, the scripts work with the forward slash (/) instead of the backslash (\), the latter being commonly used in folder paths in Windows. In addition, a forward slash is necessary at the end of folder paths (commonly missing if copying and pasting the folder path from Windows). Other specific considerations are provided below.

**Fig. 2:**
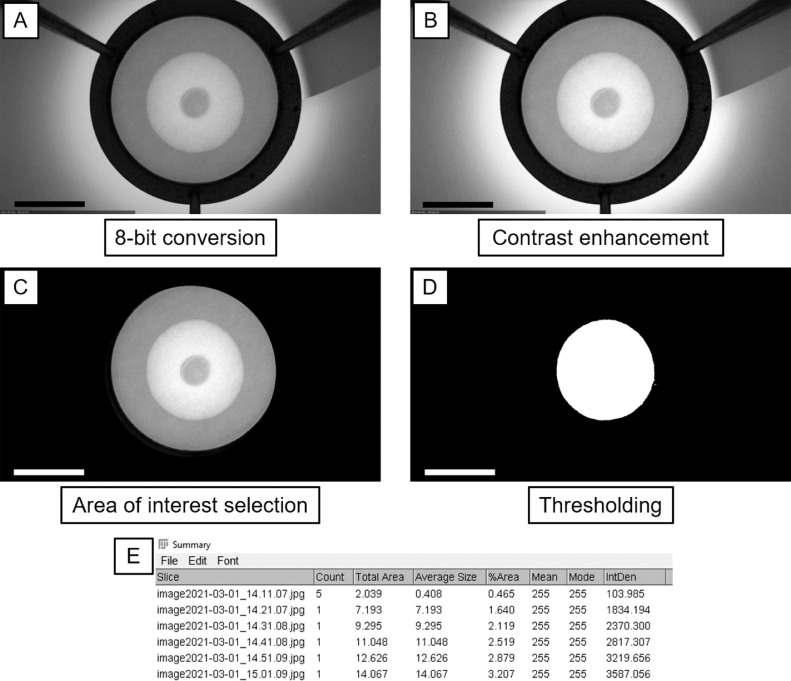
Image conversion and analysis using Script #1 and #2. (A) Conversion of the original image (Fig. 1B) to 8-bit image (grayscale) using Script #1. (B) Contrast enhancement of (A). (C) Selection of the circular area of interest and black background application. (D) Thresholding of (C) using a cutoff value of (170, 255). (E) Portion of the summary table for analyzed images showing default measurements settings. Panels (B), (C), (D), and (E) represent the sequential image analysis steps implemented in Script #2. Scale bars in (A), (B), (C), and (D) are 5 cm.

The first script (Image Conversion for Oil Release Analysis.ijm, supplementary material and Fig. 2A) automatically converted all images contained in a folder (*input*) to 8-bit grayscale images (*8-bit*) scaled down 50% of their size (*Scale…*) and saved them in another folder (*output*) as jpeg images using original filename (*saveAs*). First, we used the command *function* to group together all commands needed for each image (calling this list of commands *action*). The command *action* opened an image located in the input folder, converted it to 8-bit, scaled it down, saved it in the output folder (the image format can be changed to tiff, png, etc. if needed), and closed the image. Scaling down was performed to reduce the computing power needed to later analyze the images (from 3.5-MB full-HD images to 43-kB images). This step is optional if (i) the camera is not full HD, (ii) the total number of images to be analyzed are less than 20-30, or (iii) the computer used for image analysis has sufficient computing power. To skip the scaling-down process, one can erase the command from the script or hide it by adding // before the command itself. To automatically convert all images in a folder, first the script calculates the number of files in the folder (*getFileList(input)*) and then a loop command (*for* …) executes the command *action* for each image. After the script has finished running, the output folder will contain all converted images.

ImageJ Script #1:

input = "C:/Users/Valoppi/Desktop/MethodsX/Oil Release/Original Images/";

output = "C:/Users/Valoppi/Desktop/MethodsX/Oil Release/Converted Images/";

function action (input, output, filename){

open(input + filename);
run("8-bit");
run("Scale...", "x=0.5 y=0.5 width=960 height=540 interpolation=Bilinear average create");
saveAs("Jpeg",output + filename);
close();
}

setBatchMode(true); list = getFileList(input) for (i = 0; i < list.length; i++){

action(input, output, list[i]);
}

setBatchMode(false);

The second script (Calculation Oil Release Analysis.ijm, supplementary material, and Fig. 2B–E) automatically analyzes all images contained in a folder (*input*) to calculate the area in cm^2^ of the oil stain during the oil-release test. First, the script sets the scale (*Set Scale…*) required. Before performing this command, one needs to calculate the right scale of their images. To this aim, one can use a reference in the image to set the scale (we used the diameter of the tripod top opening, equal to 12.1 cm). We drew a straight line on the reference, recorded the length in pixels, and substituted the values in the *Set Scale...* command: *distance=*pixels of the reference, *known=*reference known length, *unit=*the length unit used (cm, mm, etc.). Then, the script improved the contrast of the images (*Enhance Contrast*) which helped also by reducing possible effects caused by light fluctuation and which maximizes the image contrast by taking the brightest and darkest areas of the picture (Fig. 2B). The script selected an area of interest (*makeOval*), and removed the outside of the area of interest (*Clear Outside*) by coloring it in black (Fig. 2C). It was important to check and possibly modify the coordinates of the area of interest. We used the oval command that is defined by four coordinates (x, y, w, h). The first two coordinates (x, y) define the center of the oval in the image and the other two (w, h) define the width and height of the oval. This set of coordinates must be modified according to the tripod top opening diameter and the distance between the camera and the filter paper on the tripod. The tripod should be fixed to the table or it should not be moved so that the same set of coordinates can be used between analyses. To determine the set of coordinates for the *makeOval* function, the oval icon in the main menu of ImageJ window was selected. Next, the oval was manually drawn on a selected image, adjusted to make it fit the tripod aperture, and coordinates were retrieved from the ImageJ window (they appear under the oval icon, in case they are not visible, the oval has to be selected in the image by clicking on it and adjust it again).

Following this, the script thresholded the image (*setThreshold*). This function set a cutoff value (threshold) for which pixels were converted to black or white. The oil stain was converted to white and the remaining part of the image was converted to black (Fig. 2D). The lower threshold value (which was set to 170, ImageJ Script #2) was set according to the illumination used during the image capture. A preliminary analysis was performed to determine the best threshold value that can select the area of the oil stain, so that the pixels belonging to the oil stain are converted to white pixels and the remaining pixels are converted to black. This can be achieved by first loading an image in ImageJ. Then, one needs to adjust the contrast (Image → Adjust → Brightness/Contrast… → Auto →Apply), and recall the threshold command (Image → Adjust → Threshold…). The threshold window appears showing a plot and two adjustable parameters (numerical values). The higher parameter is set at 255, whereas the lower parameter is variable (*e.g.,* 150). Only the lower value has to be adjusted such that the oil stain is not covered by the mask that was automatically generated (in this example the value was 170). This value must be used in the script.The same light intensity should be maintained within and between analyses, such that the same threshold values can be used for all images. This means that the light source and the distance between the light and the setup should be consistent. Finally, the script calculated the area of the white object that remained after the thresholding (*Analyze Particles...*) and summarized the calculations in a table (*summarize*) (Fig. 2E). The image was then closed. In case one wants to assess the quality of each analyzed image, the command *close()* can be erased or skipped by adding // before the command itself. This will leave the analyzed image open. To automate the analysis for all images, we used the same commands as in the first script (*getFileList* and loop command *for…*). After the script had finished running, the table called “Summary” contained all numerical values of the analysis. The table contains the name of the files, the number of objects recorded in each image, and the total area (sum of the area of each object). The table content can be modified by ticking or unticking the setting options in Analyze → Set Measurements… menu. For the oil-release analysis, only the total area displayed was of interest.

ImageJ Script #2:

input = " C:/Users/Valoppi/Desktop/MethodsX/Oil Release/Converted Images/";

function action (input, filename){

open(input + filename);
run("Set Scale...", "distance=416 known=12.1 pixel=1 unit=cm global");
run("Enhance Contrast", "saturated=0.35");
run("Apply LUT");
makeOval(265, 46, 431, 432);
setBackgroundColor(0, 0, 0);
run("Clear Outside");
setAutoThreshold("Default dark");
setThreshold(170, 255);
setOption("BlackBackground", true);
run("Convert to Mask");
run("Analyze Particles...", "size=0.005-Infinity summarize");
close();
}

list = getFileList(infor (i = 0; i < list.length; i++){

action(input, list[i]);
}

An example of image conversion (Script #1) and the steps of the analysis (Script #2) is shown in Fig. 2. The original image (Fig. 1B) was converted to a scaled down 8-bit image (Fig. 2A) using the first script. Fig. 2B–E show the step-by-step output of the second script.

**Fig. 3:**
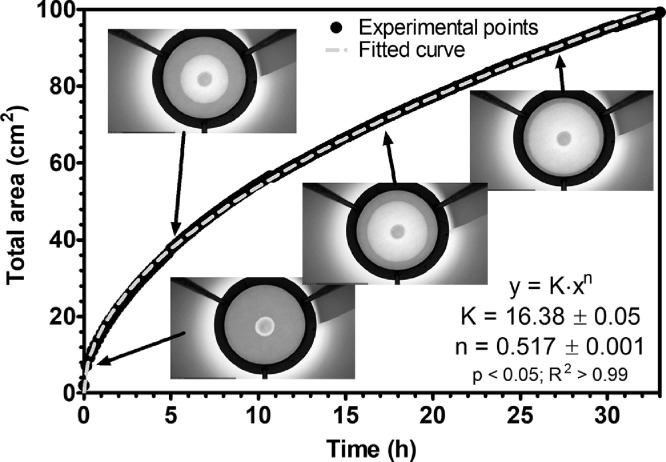
Oil-release expressed as oil-stain area as a function of time. This plot was obtained after applying both scripts to a set of 200 images automatically captured over 33 h. Images show the evolution of the oil stain at pre-selected times. The maximum total visible area is 115 cm^2^, which corresponds to the area of the filter paper inside the tripod top opening frame, calculated as the area of a circle of diameter 12.1 cm. Fitted curve following an exponential equation is shown as a dashed grey line. The equation and the results of the regression analysis are shown in the bottom right corner of the figure.

Fig. 3 shows the evolution of the oil stain area in cm^2^ on a filter paper as a function of time. Two-hundred images were analyzed using the two scripts with a processing time of 3 min to obtain the data shown in Fig. 3. Selected images were added to visually observe the evolution of the oil stain. The full sequence of images is presented in the supplementary material as a gif file (Oil Release GIF). Data were then fitted using a power law equation ($y=K\cdot x^{n}$, where *y* is the total oil stain area, *K* is the rate constant, *x* is time, and *n* is the exponent; the procedure used is described in [7]). The large number of data points coupled with the quality of the data yielded low-fitting errors on the estimated parameters (Fig. 3), which can better highlight the differences among samples when comparing different LBM oil-release curves. We then manually calculated the oil-stain area on selected images (20 images selected at regular time intervals) by measuring the diameter of the stain and assuming it to be circular (data not shown). The average difference in area between the automated method and the manual method was ±5% of the total computed area. This difference was due to the assumption that the shape of the oil stain is circular and errors in measuring the diameter of the oil stain. The oil stain was nearly circular in the set of images analyzed here. However, the stain can develop in an irregular way, possibly introducing deviations from the actual area if the assumption of a circular stain form is kept. An irregular oil-stain shape can also provide information on the preferred retention of oil within the LBM network, highlighting possible inhomogeneities in the material. Therefore, our method, which analyses the true shape of the oil stain, is more versatile than analyses using circular oil shapes, as our method makes no geometrical assumptions about the oil stain.

Finally, we assessed the repeatability of the method by measuring five samples over a period of 24 h. The error calculated for the 95% confidence interval linearly depended on time, starting from 1.5 cm^2^ at the beginning of the analysis, and ending at 6 cm^2^ at the end of the analysis. However, the relative standard deviation (RSD, *i.e.,* the percentage ratio between the standard deviation and the mean) showed an opposite behavior moving from 40% at the beginning of the analysis to 15% after 30 min, to then reach a plateau of 6-7% after 5 h of analysis. Overall, even if the RSD is high during the first part of the analysis (where the oil stain area is small), the 95% confidence interval error was within an acceptable range, showing that the method has a good repeatability.

The proposed method can process many images within a short time frame, expediting image processing for analyzing the oil release of lipid-based materials. In addition, this method can be modified or customized for other non-volatile solvent-release analyses from a gel network, based on image capturing and analysis. This method can also analyze materials which can be affected by shrinkage during analysis.

## Appendix Supplementary materials

Supplementary material *and/or* Additional information • **Oil Release GIF.** Animation showing the evolution of the oil stain observed from 200 images captured over 33 h. • **Image Conversion for Oil Release Analysis.** Macro file containing Script #1 for automatic conversion to 8-bit and size reduction of oil-release test images. • **Calculation Oil Release Analysis.** Macro file containing Script #2 for automatic analysis of oil-release test images. Image, application 1
