# Supplementary figures and images for: Automated image analysis method for oil-release test of lipid-based materials
Source: MethodsX. 2021 Jul 10;8:101447. doi: 10.1016/j.mex.2021.101447 (PMC8374682; doi:10.1016/j.mex.2021.101447)

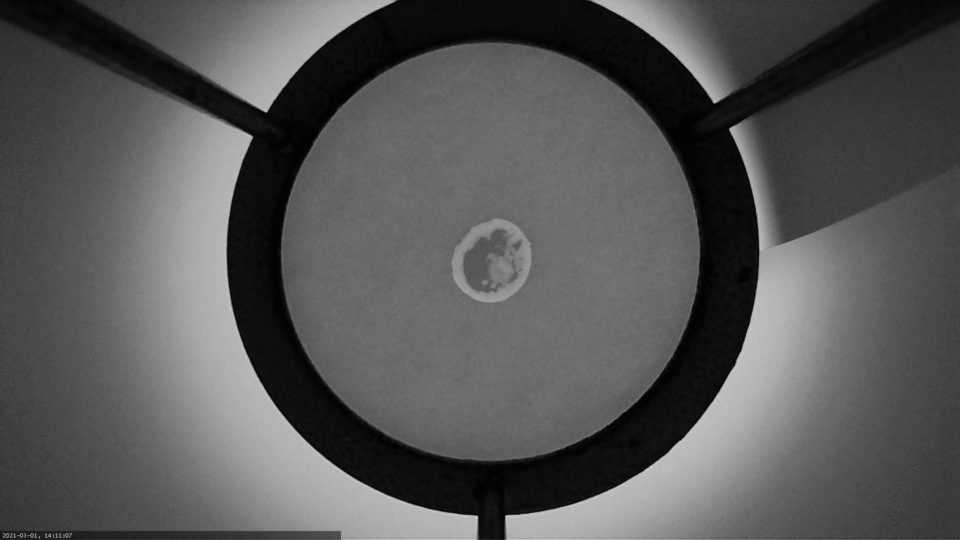

Supplement: Supplementary file 1 — Supplementary material and/or Additional information • Oil Release GIF. Animation showing the evolution of the oil stain observed from 200 images captured over 33 h. • Image Conversion for Oil Release Analysis. Macro file containing Script #1 for automatic conversion to 8-bit and size reduction of oil-release test images. • Calculation Oil Release Analysis. Macro file containing Script #2 for automatic analysis of oil-release test images. [file mmc1.zip › Oil Release GIF.gif]
